# *At5g19540* Encodes a Novel Protein That Affects Pigment Metabolism and Chloroplast Development in *Arabidopsis thaliana*

**DOI:** 10.3389/fpls.2017.02140

**Published:** 2017-12-19

**Authors:** Xing-Qi Huang, Lei Zhao, Jin-Di Rui, Chang-Fang Zhou, Zhong Zhuang, Shan Lu

**Affiliations:** State Key Laboratory of Pharmaceutical Biotechnology, School of Life Sciences, Nanjing University, Nanjing, China

**Keywords:** *Arabidopsis thaliana*, chloroplast, development, pigments, Dwarf and Yellow 1 (DY1), RABE1b

## Abstract

Chlorophylls and carotenoids not only function in photosynthesis and photoprotection but are also involved in the assembly of thylakoid membranes and the stabilization of apoproteins in photosystems. In this study, we identified a nuclear gene required for chlorophyll and carotenoid metabolism, namely, *DWARF AND YELLOW 1* (*DY1*). Growth of the loss-of-function *dy1* mutant was severely retarded, and the seedlings of this mutant accumulated significantly less amounts of both chlorophylls and carotenoids in cotyledons and rosette leaves, although genes related to pigment metabolism did not show corresponding fluctuation at the transcriptional level. In chloroplasts of the *dy1* leaves, thylakoids were loosely packed into grana. The *dy1* mutant also possessed severely impaired photosynthetic and photoprotective abilities. *DY1* encodes a chloroplast stroma protein that is highly conserved in vascular plants. Our results demonstrated that after the full-length DY1 (53 kDa) was imported into the chloroplast and its N-terminal transit peptide was processed, the C-terminal end of this premature DY1 (42 kDa) was also removed during the maturation of rosette leaves, resulting in a 24-kDa mature peptide. Our blue native PAGE and Western blot analyses showed the presence of both premature and mature forms of DY1 in protein complexes. The involvement of DY1 in chloroplast development is discussed.

## Introduction

Chloroplast development is a key event for plant growth and adaptation. It involves different processes, including the expression of nuclear and plastid genes, the biosynthesis and accumulation of chlorophylls and carotenoids, and the assembly of membrane systems, that are highly coordinated in a spatiotemporal context (Mullet, 1988; Pfalz and Pfannschmidt, 2013; Andriankaja et al., 2014). Different mechanisms of regulating chloroplast development through pigment biosynthesis have been reported. For example, members of the PHYTOCHROME-INTERACTING FACTOR (PIF) family interact with DELLA to regulate the expression of light-dependent genes, especially those genes for chlorophyll and carotenoid metabolism (Cheminant et al., 2011). The expression of the gene for phytoene synthase (PSY), a key enzyme for carotenoid biosynthesis, is also directly repressed by PIFs (Toledo-Ortiz et al., 2010). Moreover, genes involved in the assembly of thylakoid membranes and in plastid-encoded RNA polymerase (PEP)-dependent transcription, such as the DnaJ-like zinc finger domain protein PSA2 and the heat shock protein HSP21, were also reported to modulate chloroplast development and to affect acclimation to high-light stress (Zhong et al., 2013; Fristedt et al., 2014; Wang et al., 2016).

It has been demonstrated that pigment mutants with albino, yellow or variegated cotyledons or true leaves are a good system for the discovery of novel regulating components. For example, the well-studied *immutans* (*im*) and *variegated* (*var*) mutants were found to encode a plastid terminal oxidase and FtsH, respectively (Aluru et al., 2001; Sakamoto, 2003; Yu et al., 2007). Studies of *snowy cotyledon* mutants also identified the involvement of chloroplast elongation factor G (SCO1), zinc-finger domain protein (SCO2), microtubule and peroxisome-associated protein (SCO3) and proteinase (SCO4) in chloroplast development (Albrecht et al., 2006, 2008, 2010; Albrecht-Borth et al., 2013). These studies illustrated that different mechanisms at metabolic, transcriptional, and translational levels are involved in the regulation of pigment metabolism, chloroplast development, or both.

In this work, by screening a pool of *Arabidopsis thaliana* T-DNA insertion mutants for seedlings with abnormal pigment accumulation, we identified a novel gene, *Dwarf and Yellow 1* (*DY1*/*At5g19540*) that regulates both pigment metabolism and thylakoid membrane assembly.

## Materials and Methods

### Plant Materials and Growth Conditions

The *Arabidopsis thaliana* T-DNA insertion mutant pool (CS76508) was purchased from the Arabidopsis Biological Resource Center (ABRC, Ohio State University, Columbus, OH, United States). All *A. thaliana* plants used in this study were in Col-0 wild-type (WT) background. After 3 days stratification at 4°C in the dark, seeds were germinated on Murashige-Skoog (MS) plates containing 2% sucrose at 22°C under a light intensity of 120 μmol photons m^-2^ s^-1^ with a 16 h/8 h light/dark photoperiod. Two-week-old seedlings were moved to grow in soil (a mixture of peat moss, vermiculite and perlite at 1:1:1) under the same conditions (Wang et al., 2016).

Mutants were screened on MS plates containing 25 mg/L kanamycin. Seedlings with albino, yellowish, or pale green cotyledons were moved to grow in soil, and their seeds were individually collected.

### Molecular Manipulation and Plant Transformation

Genomic DNA was extracted from rosette leaves using the CTAB method (Green and Sambrook, 2012). For identifying T-DNA insertion position of each mutant, genome walking was performed using a Genome Walking Kit (TaKaRa, Shiga, Japan) following the manufacturer’s manual. Homozygous progenies of the mutants were screened according to the SIGnAL iSect tool^1^. All primers used in this study are listed in **Supplementary Table S1**.

RNA was isolated using RNAiso Plus Reagent (TaKaRa) according to manufacturer’s instruction. Total RNA (1 μg) was reverse transcribed using a PrimeScript 1st Strand cDNA synthesis Kit (TaKaRa). Transcript abundance of each gene studied was determined by quantitative real-time PCR (qPCR) in a Thermal Cycler Dice Real Time System TP800 (TaKaRa) using SYBR Premix ExTaq II (TaKaRa), following the manufacturer’s manuals, and calculated using the comparative *C*_T_ method (Schmittgen and Livak, 2008). *ACT 2* (*At3g18780*) was used as a reference. At least three biological replicates, each with three repeats, were analyzed for each sample.

For genetic complementation, a 4332 bp genomic DNA fragment of *DY1* (*At5g19540*), ranging from -2044 bp upstream of the translation initiation codon (ATG) to 290 bp downstream of the stop codon, was amplified using primers DY1-GF and DY1-GR, and cloned into the *Bam*H I site of pCAMBIA1300 (CAMBIA, Canberra, ACT, Australia) to generate the construct *DY1:DY1*. For subcellular localization study, full-length open reading frame (ORF) of *DY1* or *At4g20360* was amplified from the 1st strand cDNA pool using primers DY1-HF and DY1-ER for *DY1*, or RABE1b-HF and RABE1b-ER for *At4g20360*, and subsequently cloned into the *Bam*H I site of pA7-eYFP to generate the construct *35S:DY1-eYFP* or *35S:At4g20360-eYFP*. To generate the construct for expressing DY1 with dual fluorescent proteins, the coding sequences for the chloroplast transit peptide (cTP) of DY1 (cTP^DY 1^) (using primer pair DY1-cTP-HF and DY1-cTP-ER), eYFP (eYFP-HF and eYFP-ER), DY1 without cTP (DY1^ΔcTP^) (DY1-ΔcTP-HF and DY1-ΔcTP-ER) and mCherry (mCherry-HF and mCherry-ER), were separately amplified and joined together into the *Bam*H I and *Sac* I sites of pA7-eYFP using In-Fusion technique (TaKaRa). The entire fragment encoding the cTP^DY 1^-eYFP-DY1^ΔcTP^-mCherry fusion protein was further amplified (DY1-mature-HF and DY1-mature-ER) and then subcloned into the *Nco* I site of pCAMBIA1300-RTL2 using In-Fusion technique for transient expression in tobacco leaves and genetic transformation of Arabidopsis. *A. thaliana* was transformed by the floral dip method (Clough and Bent, 1998).

For transient transformation, protoplasts were isolated from rosette leaves according to Yoo et al. (2007). About 20 μg of purified plasmid DNA was applied for each transformation. The protoplasts were incubated in the dark at 22°C overnight before microscopy observation.

For all PCR amplifications, high-fidelity PrimeSTAR DNA polymerase (TaKaRa) was used according to the manufacturer’s instruction.

### Sequence Analysis

Subcellular localization of DY1 were predicted using online programs ChloroP and TargetP (Emanuelsson et al., 1999; Emanuelsson et al., 2007). Deduced amino acid sequence of DY1 was used as a query to search its homologs in different plant species of which full genomes have been sequenced in GenBank using the BlastP algorithm. Sequences were aligned using the ClustalX program and a maximum-likelihood phylogenetic tree was constructed using MEGA 6 with a bootstrap replication value of 1,000 (Chenna et al., 2003; Tamura et al., 2013).

### Northern Blot

To perform Northern blot, total RNA was extracted from leaves of 3-week-old *dy1* mutant and WT plants. Ten microgram of total RNA was loaded for each lane and separated on 1.5% agarose gel. After separation, the RNA was blotted onto a positively charged nylon membrane (GE Healthcare, Pittsburgh, PA, United States). The probe for detecting *DY1* transcripts was amplified using primer pair DY1-Probe-F and DY1-Probe-R, with *DY1* cDNA as a template, and then labeled using DIG DNA Labeling Mix (Roche, Basel, Switzerland). Probing, washing, and detection of *DY1* were performed according to the DIG High Prime DNA Labeling and Detection Starter Kit II user’s manual (Roche).

### Protein Extraction and Western Blot Analysis

Total protein was extracted from rosette leaves using the trichloroacetic acid (TCA) precipitation method. Approximately 1 g of rosette leaves was homogenized in liquid nitrogen. Ten milliliter of cold 10% TCA in acetone was then added and mixed well by vortex and sonication. The mixture was incubated at -20°C for at least 4 h and then centrifuged at 4°C, 15,000 *g* for 20 min. The pellet was washed several times with cold acetone, air dried briefly, and solubilized in 9 M urea (in 100 mM phosphate buffered saline, 1 mM DTT) with sonication. The precipitation was repeated once, and the pelleted protein was finally dissolved in 9 M urea solution for direct use or storage.

Protein samples were mixed with equal amounts of 2 × SDS loading buffer (Green and Sambrook, 2012), heated at 65°C for 10 min, separated on 12 % SDS–polyacrylamide gel and subsequently blotted onto nitrocellulose membrane (GE Healthcare) for immunodetection. A peptide (WSIRNPTDLETSSY) was synthesized based on deduced amino acid sequence of DY1 and used as an antigen to immune rabbits by GenScript (Nanjing, China). Antiserum was purified from blots according to Harlow and Lane (1988). Antibody against Actin 11 was purchased from Agrisera (Vännäs, Sweden). Horseradish peroxidase (HRP)-conjugated secondary antibody against rabbit IgG was from Promega (Madison, WI, United States). Common protocols (Green and Sambrook, 2012) and the manufacturers’ manuals for electrophoresis, semi-dry blotting and Western detection using the ECL Western Blotting Substrate (Promega, Madison, WI, United States) were followed.

### Microscopy

A FluoView FV1000 (Olympus, Tokyo, Japan) laser scanning confocal microscopy system was used for fluorescence observation. The eYFP fluorescent was excited with 488 nm laser and the emitted light was recorded from 500 to 530 nm. The mCherry fluorescent was excited with 543 nm laser, recorded from 580 to 620 nm. 543 nm laser excitation and 680 to 720 nm recording range were used for chlorophyll auto-fluorescence observation.

For transmission electron microscopy (TEM) analysis, leaves from 3-week-old seedlings were fixed, embedded and sectioned according to Faso et al. (2009). A Hitachi-7700 transmission electron microscope (Hitachi, Tokyo, Japan) was used for observation and image capturing.

### Chloroplast Isolation, Fractionation and Blue Native (BN)-PAGE

About 10 g of rosette leaves were homogenized briefly in cold chloroplast isolation buffer (0.3 M sorbitol, 5 mM MgCl_2_, 5 mM EGTA, 5 mM EDTA, 20 mM HEPES, 10 mM NaHCO_3_, pH 8.0) and then filtered through two layers of miracloth (EMD Millipore, Billerica, MA, United States). Crude chloroplasts were collected by centrifugation for 5 min at 4°C, 1,000 *g*. After the separation of crude chloroplasts on a 2-layer Percoll gradient (40 and 80%), intact chloroplasts were obtained from the interface between two layers. Purified chloroplasts were further lysed in TE solution (10 mM Tris–HCl, 1 mM EDTA, pH 7.5) for 30 min on ice, followed by a centrifugation for 5 min at 3,000 *g* to collect thylakoid membranes.

For BN-PAGE, intact chloroplasts were solubilized in Solubilization Buffer (50 mM Bis–Tris, pH 7.0, 0.5 M aminocaproic acid, 10% glycerol, 1% *n*-dodecyl β-D-maltoside and 1 mM PMSF) on ice for 10 min before separation on a 4% ∼ 14% gradient gel. After the first dimension electrophoresis, the gel was either directly blotted onto a PVDF membrane (Millipore) or further separated by SDS–PAGE for a second dimension (Zhou et al., 2017).

### Pigment Quantification

Chlorophylls and carotenoids were isolated from rosette leaves according to Pogson et al. (1996). One hundred milligram of leaves was ground in liquid nitrogen into fine powder and mixed thoroughly with 250 μl 80% acetone containing 10 μg deuteroporphyrin IX dimethyl ester (Sigma–Aldrich, St. Louis, MO, United States) as an internal standard. The extract was then mixed with 250 μl ethyl acetate, followed by 200 μl of water. After a centrifugation at 4°C, 15,000 *g* for 10 min, the upper organic phase was collected and dried under a stream of nitrogen. The pigment was re-dissolved in Solvent A (acetonitrile:water:trimethylamine = 9:1:0.01) for high-performance liquid chromatography (HPLC) analysis. All steps were carried out under dim light.

Total pigment samples were analyzed by HPLC (Waters 2695 separation module) with a Waters ODS2 C18 analytical column (5 μm, 4.6 mm × 250 mm) and 2998 photodiode array detector (Waters, Milford, MA, United States) according to Wang et al. (2016).

### Chlorophyll Fluorescence Analysis

Chlorophyll fluorescence of 3-week-old *dy1* mutant and WT plants were measured by a MINI-PAM fluorometer (Walz, Effeltrich, Germany) according to Wang et al. (2016). For light-response curves of PSII quantum yield (ΦPSII) and non-photochemical quenching (NPQ), dark-adapted plants were illuminated at a series of photosynthetically active photon flux densities (PPFD) (3, 19, 43, 145, 300, 387, 611, 1,006, and 1,312 μmol photons m^-2^ s^-1^). The minimal yield of fluorescence (*F*_0_) under 650 nm was measured at 0.8 μmol photons m^-2^ s^-1^. To estimate the maximum fluorescence yield (*F*_m_), a saturating pulse (0.8 s, 5,000 μmol photons m^-2^ s^-1^) was applied.

### Yeast Two-Hybrid Screening

The yeast two-hybrid screening was performed largely according to the Matchmaker^TM^ GAL4 Two-Hybrid System 3 & Libraries User Manual (TaKaRa). Full length ORF of *DY1* was cloned into pDEST32 (Invitrogen, Carlsbad, CA, United States) and then transformed into the yeast (*Saccharomyces cerevisiae*) strain AH109. *A. thaliana* cDNA library constructed in pDEST22 (Invitrogen) was maintained in the yeast strain Y187. After mating, diploid yeast cells were screened on SD/-Leu/-Trp/-His triple dropout medium. From positive colonies, cDNA insertions encoding the prey proteins were amplified by PCR and sequenced. Candidate genes were further tested for pair-wise interaction using a pGAD-T7/pGBK-T7 system (TaKaRa). Briefly, the coding sequences of *DY1* and candidate genes were cloned into pGAD-T7 and pGBK-T7, respectively. Plasmids were co-transformed into yeast strain AH109 and selected on SD/-Leu/-Trp double drop out plates. Colonies were serial diluted and further tested on SD/-Leu/-Trp/-His/-Ade quadruple dropout plates for detecting protein–protein interactions.

### Statistical Analysis

Statistical significance was tested using GraphPad Prism6 (GraphPad Software). Data are shown as the means ± SD of at least three replications.

## Results

### Characterization of the *dy1* Mutant

**FIGURE 1:**
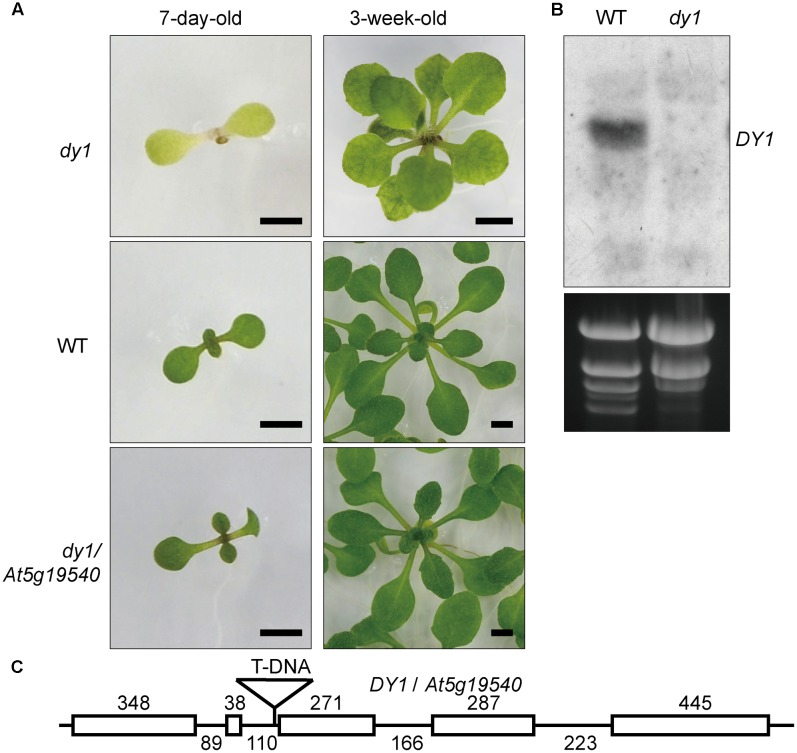
Phenotype and characterization of the *dy1* mutant plant. **(A)** *dy1*, WT and *At5g19540* transformed *dy1* plants grown on MS plates. Bars = 2 mm. **(B)** Northern blot showing the silence of *At5g19540* in the *dy1* mutant. **(C)** T-DNA insertion position of *dy1*. The insertion is 3 bp upstream the third exon of *At5g19540*. Sizes of each intron and exon are labeled.

We screened 10,000 individuals of the T-DNA insertion mutant library. At least 20 lines showed pale green, yellowish or completely albino cotyledons. The T-DNA insertion positions of these lines were identified by genome walking. One of these lines that showed dwarfed growth and yellowish cotyledons (**Figure 1A**) was proved to harbor a T-DNA insertion in front of the third exon of *At5g19540* (**Figure 1C**). We named this uncharacterized gene *Dwarf and Yellow 1* (*DY1*). Northern blot (**Figure 1B**) and genomic complementation (**Figure 1A**) experiments confirmed that *At5g19540* is responsible for the corresponding phenotypes. *DY1* encodes a 53-kDa protein with a predicted N-terminal transit peptide (cTP), which was predicted to target DY1 to chloroplasts (**Supplementary Figure S1**). The *dy1* mutant seedlings were defective in de-etiolation, i.e., their cotyledons stayed yellow after illumination. Vegetative growth of the *dy1* seedlings was severely retarded. Juvenile true leaves of the *dy1* mutant also had defects in their pigmentation, whereas mature leaves of the mutant were greener (**Figure 1A**).

### *DY1* Is Highly Conserved in Plants

**FIGURE 2:**
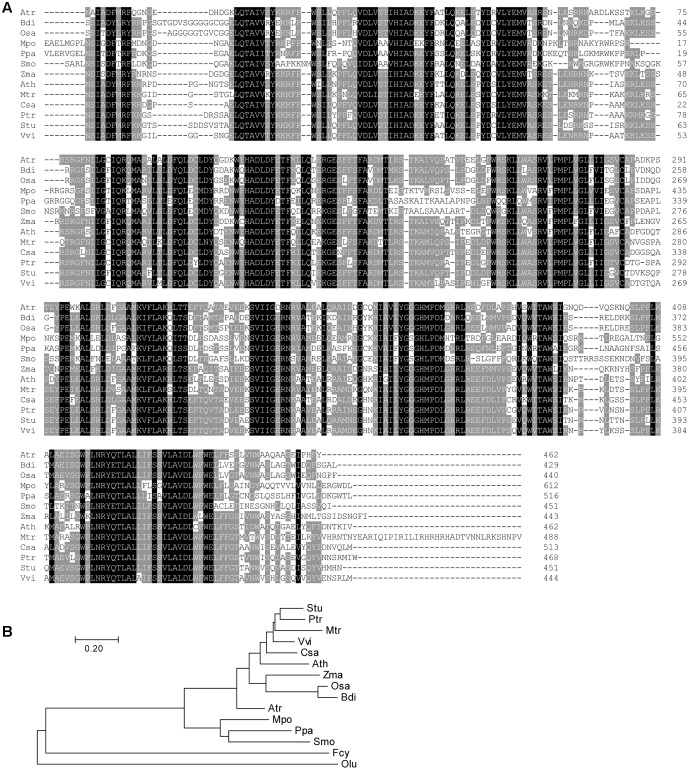
Multiple sequence alignment **(A)** and phylogenetic analysis **(B)** of DY1 homologs. Sequences are from plant species for which full genomes have been sequenced, including *Amborella trichopoda* (Atr, XP_006833290.3), *Arabidopsis thaliana* (Ath, OAO95398.1), *Brachypodium distachyon* (Bdi, XP_003574414.1), *Cucumis sativus* (Csa, XP_004150055.2), *Marchantia polymorpha* (Mpo, OAE23078.1), *Medicago truncatula* (Mtr, AFK48173.1), *Oryza sativa* (Osa, XP_015650923.1), *Physcomitrella patens* (Ppa, XP_001768011.1), *Populus trichocarpa* (Ptr, XP_002324991.1), *Selaginella moellendorffii* (Smo, XP_002961080.1), *Solanum tuberosum* (Stu, XP_006353458.1), *Vitis vinifera* (Vvi, XP_002280972.1), *Zostera marina* (Zma, KMZ73824.1). Sequences from the green alga *Ostreococcus lucimarinus* (Olu, XP_001420303.1) and the diatom *Fragilariopsis cylindrus* (Fcy, OEU12403.1) which showed similarities with DY1 were incorporated in the phylogenetic tree. The scale bar corresponds to 20% amino acid sequence divergence.

We did not find functional characterization reports nor annotation for *DY1*, and therefore, we searched GenBank for homologs of *DY1*. In each plant species for which a full genome has been sequenced, only one copy of a *DY1* homolog gene was found. We did not find any close homologs of *DY1* in cyanobacteria, green algae or diatoms, although there are some sequences in certain organisms with weak similarities to *DY1* (**Figure 2**).

Comparisons of the deduced amino acid sequences of DY1 homologs identified highly conserved regions spanning the entire sequence beyond the homologs’ cTPs (**Figure 2A**). Overall sequence identity among DY1 homologs is higher than 40%, and it is over 60% if we exclude the most variable cTP regions. On a phylogenetic tree, DY1 homologs formed two major clades, one of which was composed of members from angiosperms and the other of which consisted of homologs from the remaining land plants, including gymnosperms and bryophytes. The angiosperm clade can be further divided into 2 sub-clades, one each for dicots and monocots (**Figure 2B**).

### The *dy1* Mutant Is Impaired in Pigment Biosynthesis and Light Acclimation

**FIGURE 3:**
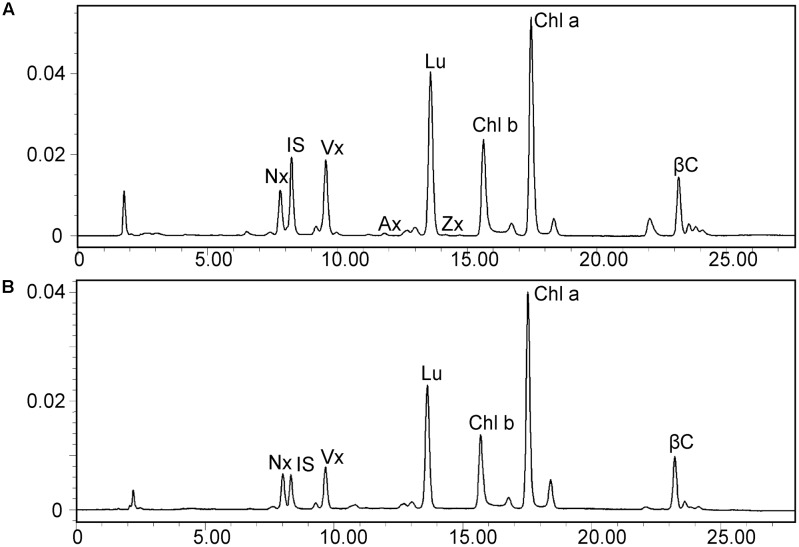
High-performance liquid chromatography (HPLC) separation of pigments in 3-week-old rosette leaves of the *dy1* mutant **(A)** and the wild-type **(B)** plants. Peaks are neoxanthin (Nx), internal standard (IS), violaxanthin (Vx), antheraxanthin (Ax), lutein (Lu), zeaxanthin (Zx), β-carotene (βC), and chlorophyll *a* and *b* (Chl *a* and Chl *b*).

Leaves of the *dy1* mutant showed a distinctly lighter color compared to those of the WT plants. Therefore, we postulated that the *dy1* mutant might accumulate lower amounts of pigments. In our quantification of the pigments in 3-week-old seedlings, both chlorophyll *a* and *b* contents in the *dy1* mutant were significantly lower than in the WT plants. For carotenoids, *dy1* leaves also accumulated less neoxanthin, violaxanthin, lutein and β-carotene compared with the WT plants. However, under normal growth conditions, two xanthophylls, antheraxanthin and zeaxanthin, were found to accumulate in *dy1* leaves, although at trace amounts (**Figure 3**). The quantities of all pigment contents are listed in **Table 1**.

**Table 1:** Leaf pigment profile of the *Arabidopsis thaliana* wild-type (WT) and *dy1* mutant plants (μg/g fresh weight).

Pigment	WT	*dy1*	Ratio (*dy1*/WT)
Chl *a*	835.03 ± 6.82	289.55 ± 21.61^∗∗^	34.6%
Chl *b*	325.91 ± 14.33	128.98 ± 0.20^∗∗^	39.6%
Lutein	208.06 ± 0.21	77.46 ± 3.06^∗∗^	37.0%
β-Carotene	76.31 ± 2.56	24.86 ± 2.06^∗∗^	32.6%
Neoxanthin	51.61 ± 2.80	17.85 ± 0.11^∗∗^	34.5%
Violaxanthin	60.62 ± 0.45	34.18 ± 0.99^∗∗^	56.4%
Antheraxanthin	–	0.98 ± 0.08^∗∗^	
Zeaxanthin	–	0.711 ± 0.04^∗∗^	


*Data represent means ± SD (Student’s *t*-test; *n* = 6; ^∗∗^*P* < 0.01).*

**FIGURE 4:**
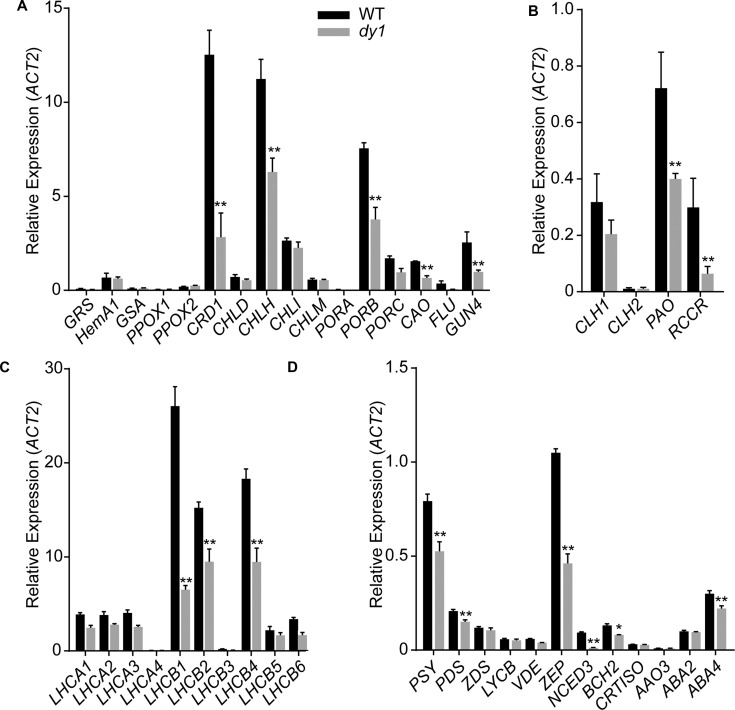
Expression of the genes encoding enzymes for chlorophyll biosynthesis **(A)** and turnover **(B)**, chlorophyll-binding proteins **(C)** and enzymes for carotenoid metabolism **(D)** in rosette leaves of the 3-week-old *dy1* and wild-type (WT) seedlings. Transcript abundance of each gene was determined by quantitative real-time PCR and are normalized against the level of *ACT2* (*At3g18780*). Data represent means ± SD (Student’s *t*-test; *n* = 3; ^∗^*P* < 0.05; ^∗∗^*P* < 0.01).

We then quantified transcript abundances of genes for chlorophyll and carotenoid biosynthesis. In 3-week-old *dy1* leaves, the expression of most of the genes for chlorophyll biosynthesis, e.g., *CRD1*, *CHLH*, *PORB*, *GUN4* and *CAO*, were down-regulated (**Figure 4A**). Interestingly, genes for chlorophyll turnover, including *PAO* and *RCCR*, were also down-regulated in *dy1* (**Figure 4B**). Moreover, the expression of most of the genes for chlorophyll *a*/*b*-binding proteins (*CABs*) was also down-regulated in *dy1* (**Figure 4C**). Most of the genes for carotenoid biosynthesis and catabolism were down-regulated in *dy1* as well (**Figure 4D**). In brief, no genes for pigment metabolism were up-regulated in *dy1* in our determination.

**FIGURE 5:**
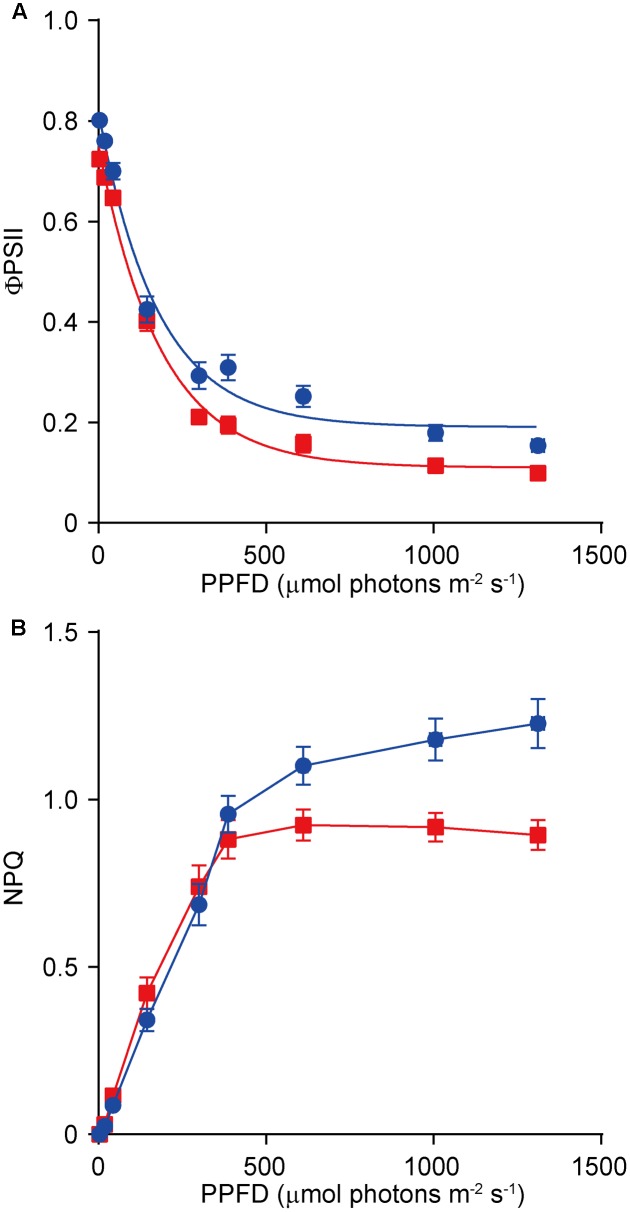
Light-response curves of PSII quantum yield (ΦPSII) **(A)** and non-photochemical quenching (NPQ) **(B)** in the wild-type (WT, in blue) and the *dy1* mutant (in red) seedlings. The measurements were taken at photosynthetic active photon flux densities (PPFD) of 3, 19, 43, 145, 300, 387, 611, 1,006, and 1,312 μmol m^-2^ s^-1^. Data represent means ± SD (*n* = 6).

Because *dy1* had lower amounts of leaf pigments, we postulated that it might also have lower photosynthetic capacity. This was confirmed by measurements of chlorophyll fluorescence parameters. In our study, *F*_v_/*F*_m_, which reflects the efficiency of PSII photochemistry, was reduced from 0.83 in the WT plants to 0.73 in *dy1* (**Table 2**). We also measured light-responsive curves to assess ΦPSII and NPQ of both *dy1* and WT plants under a series of light intensities. WT plants had higher ΦPSII than did *dy1* plants under all of the light intensities that we measured (**Figure 5A**). However, *dy1* had a higher NPQ than did the WT plants when light intensity was lower than 300 μmol photons m^-2^ s^-1^ (*P* < 0.05 at 43 and 145 μmol photons m^-2^ s^-1^) (**Figure 5B**).

**Table 2:** Chlorophyll fluorescence parameters in the leaves of the *dy1* mutant and the wild-type (WT) plants grown under 100 μmol m^-2^ s^-1^.

	WT	*dy1*
*F*_0_	199.30 ± 3.60	245.80 ± 17.00^∗∗^
*F*_m_	1156.50 ± 32.00	905.40 ± 126.00^∗∗^
*F*_v_/*F*_m_	0.8273 ± 0.0061	0.7256 ± 0.0209^∗∗^


*Data represent means ± SD (Student’s ox*t*-test; *n* = 6; ^∗∗^*P* < 0.01).*

### DY1 Affects Thylakoid Membrane Assembly

**FIGURE 6:**
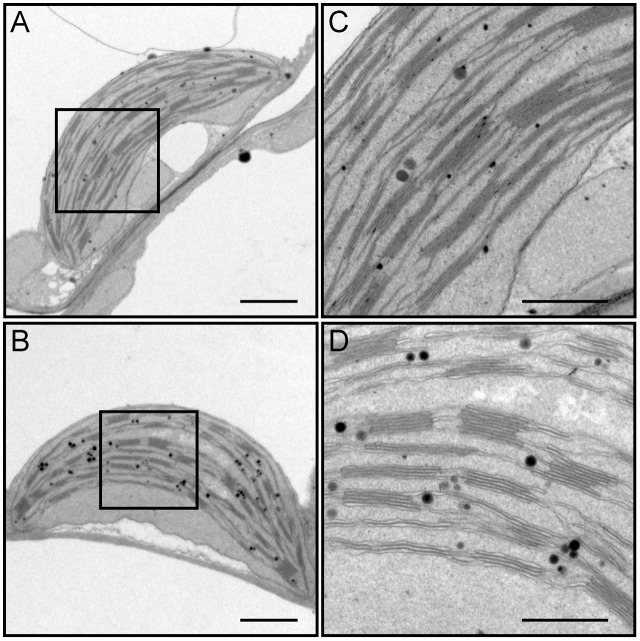
Transmission electron microscopy (TEM) observation of chloroplasts from leaves of the wild-type (WT) **(A,C)** and the *dy1* mutant seedlings **(B,D)**. Bars = 1 μm (in **A,B**) or 500 nm (in **C,D**). **(C,D)** Are magnified views of the boxed regions in **(A,B)**, respectively.

We dissected true leaves of 3-week-old *dy1* mutant and WT plants for TEM observations. The *dy1* mutant generally showed normal chloroplast shape and thylakoid formation. However, in the *dy1* mutant, thylakoid membranes were loosely packed into grana (with an average thickness of each grana thylakoid layer at 21.3 ± 1.9 nm, comparing with the WT level of 14.3 ± 1.4 nm) and possessed more plastoglobules (**Figure 6**).

### DY1 Is a Chloroplast Protein That Undergoes Post-translational Regulation

**FIGURE 7:**
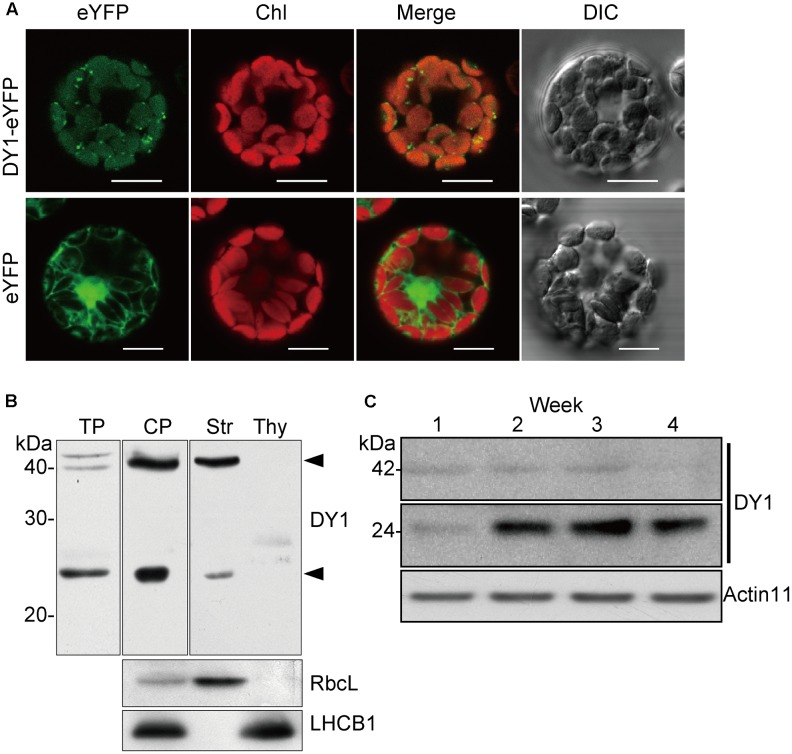
DY1 is a chloroplast stroma protein. **(A)** Subcellular localization of DY1 in chloroplast. Protoplasts transformed with pA7-eYFP-DY1 were observed under a FLUOVIEW FV1000 Laser Confocal Microscopy System (Olympus) (Bars = 20 μm). **(B)** Western blot analysis demonstrating the stroma localization of DY1. Purified chloroplast were sub-fractionated into the stroma and thylakoid membrane fractions, separated by SDS–PAGE, blotting and probed with the antiserum against DY1. LHCB1 and RbcL were probed as controls. The bands of processed and mature forms of DY1 at 42 and 24 kDa, respectively, are indicated. TP, plant total protein; CP, intact chloroplast protein; Str, stroma fraction; Thy, thylakoid membrane fraction. **(C)** Western blot analysis of the accumulation of the 24 kDa form of DY1 during leaf development. The numbers above each lane indicate the growth stages in weeks.

*DY1* encodes a protein with 462 amino acids. Both online programs ChloroP and TargetP predicted chloroplast localization. To confirm chloroplast localization, full-length DY1 protein with an enhanced yellow fluorescent protein (eYFP) fused to its C-terminus was transiently expressed in Arabidopsis protoplasts. The fluorescence of DY1-eYFP strictly overlapped with chlorophyll auto-fluorescence, indicating chloroplast localization (**Figure 7A**). From the amino acid sequence of DY1, we identified a twin-arginine (R^84^R^85^) motif (**Supplementary Figure S1**), which indicates that DY1 is probably imported into chloroplasts through the ΔpH-dependent pathway (Robinson and Bolhuis, 2001). It was reported that, for proteins imported through this pathway, their C-terminal fragments prior to the twin-arginine motif are to be cleaved after importing (Williams et al., 2000; Robinson and Bolhuis, 2001). Therefore, we isolated intact chloroplasts from WT leaves, sub-fractioned them into stroma and thylakoid fractions, and performed SDS–PAGE and Western blot analyses. From our immunoblots probed with a specific antibody raised against DY1, a band at 42 kDa was found in the total-chloroplast protein sample and the stromal fraction (**Figure 7B**). This finding agreed with the calculated size of DY1 after the cleavage (DY1^ΔcTP^) and proved the stroma localization of DY1.

**FIGURE 8:**
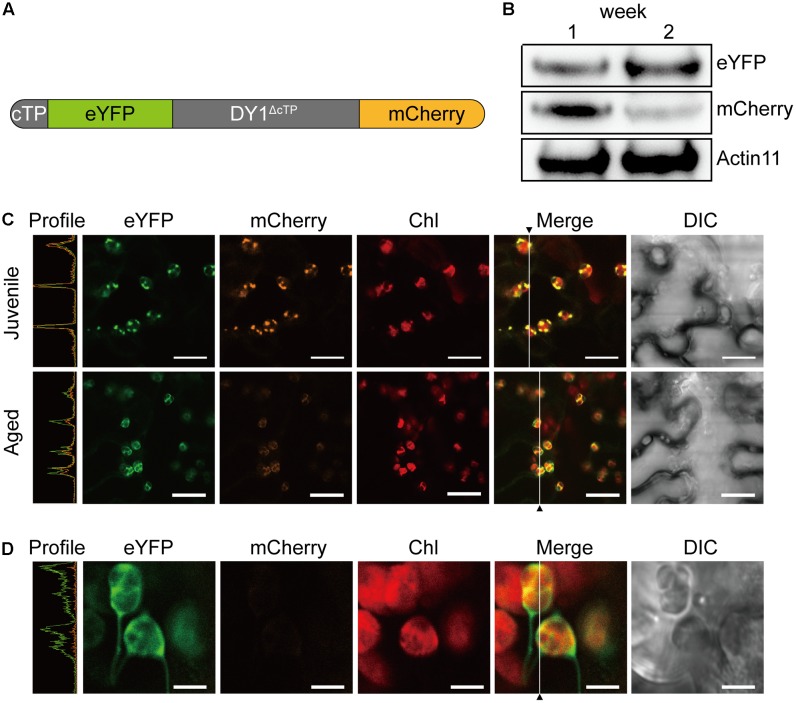
Mature form of DY1 is further processed during the development of leaves. **(A)** A diagram showing the pre-protein structure of the DY1 protein labeled with dual fluorescent proteins. **(B)** Western blot analysis of the quantities of both ends of DY1 labeled with dual fluorescent proteins. Total proteins were extracted from 1- and 2-week-old transgenic Arabidopsis seedlings. **(C)** Transient expression of the eYFP-DY1-mCherry fusion protein in tobacco leaves demonstrating a possible cleavage of the C-terminus of DY1 during leaf development. Bars = 10 μm. **(D)** Fluorescent observation of 2-week-old Arabidopsis seedlings showing the lack of mCherry signals. Bars = 5 μm. In **(C)** and **(D)**, the Profile panels (green line, eYFP signal; orange line, mCherry signal) demonstrate the signal intensity profiles along the vertical lines indicated in the Merge panels.

To our surprise, an additional smaller band of 24 kDa was frequently detected in the stroma fractions of the WT plants (**Figure 7B**). We found that the protein level of DY1^ΔcTP^ was more stable throughout developmental stages, while the content of the smaller form tended to accumulate over time (**Figure 7C**). Thus, we postulated that after being imported into chloroplasts, DY1 might be further processed during the maturation of leaves. To test this visually, we fused eYFP immediately after the cTP and mCherry at the end of the C-terminus of DY1 (**Figure 8A**), and we expressed this fusion protein with dual fluorescent tags in tobacco and Arabidopsis. In tobacco, we performed transient expression in juvenile leaves that were close to the apical shoot and in mature leaves from the bottom of the plant. After 3 days, signals of both eYFP and mCherry could be clearly observed within chloroplasts in juvenile leaves. However, in mature leaves, most observed chloroplasts had similar strengths of the eYFP signal but their mCherry signals were much weaker (**Figure 8C**). In Arabidopsis plants that were genetically transformed to express this fusion protein, it was clear that only eYFP signal could be detected in mature leaves (**Figure 8D**). When we probed the protein samples of 1- and 2-week-old transgenic Arabidopsis seedlings, antibody against eYFP could detect the DY1 fusion protein in both samples, whereas that against mCherry showed only a faint band on the blot, suggesting a removal of the C-terminus from DY1 (**Figure 8B**). These results demonstrated that DY1 matures with leaf development.

**FIGURE 9:**
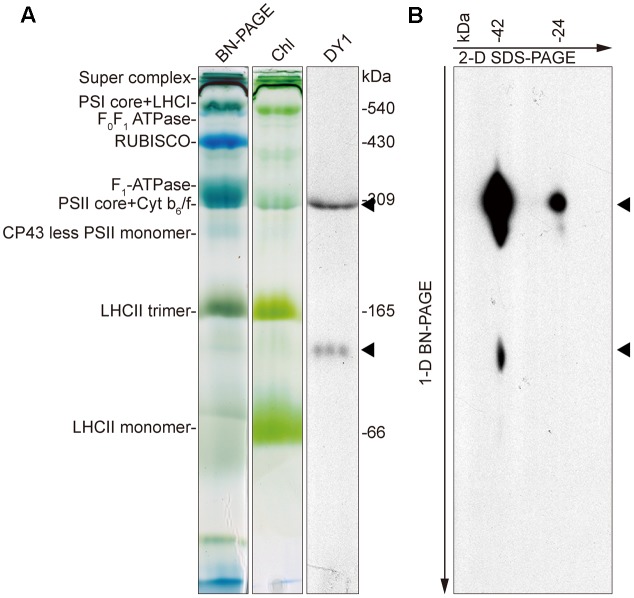
Blue-native PAGE and Western blot showing that DY1 exists in different protein complexes in chloroplast. Protein samples of intact chloroplasts from 3-week-old wild-type Arabidopsis plants were separated by BN-PAGE as the first dimension **(A)**. Chl, gel after blotting which contains chlorophylls only. The gel was either directly blotted and probed with the anti-DY1 antibody or further separated by SDS–PAGE as the second dimension and then blotted and probed with the same antibody **(B)**. Positions of DY1 protein probed by its antibody were indicated by triangles.

To determine the distributions of the two forms of DY1 in chloroplasts, we performed BN-PAGE to separate different protein complexes in chloroplasts (**Figure 9A**), followed by SDS–PAGE as a second dimension (**Figure 9B**). After electrophoresis, gels of each dimension were blotted and then probed with an antibody against DY1. Our Western blot clearly showed that both the 42- and 24-kDa forms of DY1 could be found at the position of about 300-kDa, which is around the PSII core + Cyt *b*_6_*f* complex (Timperio et al., 2007; Chen et al., 2010), and the 42-kDa form could also be probed at the position of approximately 100 kDa (**Figure 9B**).

## Discussion

In this study, by screening a pool of T-DNA insertion mutants of Arabidopsis, we identified a novel protein encoded by *At5g19540*, DY1, that is essential for the biosynthesis of both chlorophylls and carotenoids and is involved in the assembly of grana thylakoids. The fact that we failed to obtain close homologs from cyanobacteria, green algae and diatoms indicated that *DY1* was probably acquired by only the ancestor of vascular plants. The presence of only one copy of *DY1* in the genome of each plant species and the high sequence identity among its homologs suggested its critical function.

A distinct phenotype of the *dy1* mutant is its lower amounts of both chlorophylls and carotenoids. Based on our quantification of genes encoding enzymes for chlorophyll and carotenoid metabolism, both involved in biosynthesis and catabolism, and for the chlorophyll-binding proteins, it seems that the expression of all of these genes was down-regulated by the silencing of *DY1*. However, from our HPLC analysis, we found an abnormal accumulation of two xanthophylls, antheraxanthin and zeaxanthin, in *dy1* leaves under growth light. Both antheraxanthin and zeaxanthin are members of a non-photochemical, energy-quenching mechanism utilized by higher plants under high-light stress, termed the xanthophyll cycle (Demmig-Adams and Adams, 1996; Johnson et al., 2008). These pigments are absent in WT plants adapted to normal growth light, but they accumulate when there is a defect in photosystem functionality or the plants are suffering from high-light damage. Interestingly, in our analysis of chlorophyll fluorescence, the *dy1* mutant leaves showed an increase in minimal fluorescence yield (*F*_0_), together with a decreased level of the maximum fluorescence yield (*F*_m_) and the resulting maximum quantum yield of PSII (*F*_v_/*F*_m_ = [*F*_m_*–F*_0_]/*F*_m_). This indicated either a defect in electron transfer within PSII and/or a partial disconnection of the LHCII antenna, similar to the *hhl1* and *pam71* mutants (Jin et al., 2014; Schneider et al., 2016). This was supported by our determination of ΦPSII and NPQ, which were all lower in *dy1* plants compared with the WT plants. However, the *dy1* plants had a higher NPQ, comparing with the WT plants, when light intensity was lower than 300 μmol photons m^-2^ s^-1^. It is probably because of the accumulation of both zeaxanthin and antheraxanthin in *dy1* plants. Taken together, our results demonstrated the stressed condition of *dy1* PSII. This might provide an explanation of the lower amounts of total chlorophylls and carotenoids and the retarded growth of *dy1*.

The involvement of DY1 in the function of chloroplasts is further supported by our TEM observation, together with the BN-PAGE and Western blot analyses. It has been reported that grana stacking is critical for xanthophyll cycle-dependent NPQ (Goss et al., 2007). Although chloroplasts of the *dy1* mutants retained the normal shape of the WT ones, it was clear that thylakoids were loosely stacked into grana in *dy1* chloroplasts. Our Western blots showing that DY1 exists in a protein complex co-migrates with the PSII core + Cyt *b*_6_*f* complex suggest the possible involvement of DY1 in PSII.

A special feature of DY1 is its maturation. From our Western blot and observation of subcellular localization of the fusion protein with dual fluorescent tags, it is clear that the DY1 peptide is processed twice after translation. It is common for a chloroplast protein encoded by a nuclear gene to have its transit peptide removed after the pre-protein is imported into chloroplasts. But, the C-terminal end of DY1 was further cleaved during the maturation of leaves. It is unclear whether this mature form of DY1 is needed for or is simply a result of leaf maturation. Moreover, although DY1 was only found in the stroma fraction of chloroplasts, our Western blot of the BN-PAGE separated samples also demonstrated its existence in two different protein complexes, suggesting its possible interactions with different protein partners. In a yeast two-hybrid screening, we identified that DY1 interacts with RABE1b, a GTP-binding elongation factor (**Supplementary Figure S2A**). Our protoplast transient expression also confirmed chloroplast localization of RABE1b (**Supplementary Figure S2B**), indicating spatial co-localization of these two proteins. In depth analysis of the protein–protein interaction between DY1 and RABE1b might help to decipher novel machinery that regulates pigment metabolism and chloroplast functions during plant development.

## Author Contributions

X-QH, LZ, and SL conceived and designed the experiments. X-QH, LZ, J-DR, C-FZ, and ZZ performed the experiments. X-QH, LZ, C-FZ, and SL analyzed the data. SL wrote the paper.

## Conflict of Interest Statement

The authors declare that the research was conducted in the absence of any commercial or financial relationships that could be construed as a potential conflict of interest.

## Footnotes

1 http://signal.salk.edu/tdnaprimers.2.html

## Supplementary Material

FIGURE S1 Nucleotide and deduced amino acid sequence of DY1. Predicted cleavage site after the chloroplast transit peptide is indicated by a black arrow. Peptide fragment used for raising antiserum against DY1 is underlined.

FIGURE S2 DY1 interacts with a RAB GTPase homolog protein At4g20360. **(A)** Yeast two-hybrid analysis of the interaction between DY1 and At4g20360. *DY1* and *At4g20360* were cloned into pDEST32 and pDEST22, respectively. Yeast AH109 cells were co-transformed with a combination of these plasmids, and plated onto non-selective (SD/-Leu/-Trp, DDO) and selective (SD/-Leu/-Trp/-His/-Ade, QDO) plates in series dilution. **(B)** At4g20360 also localizes in chloroplasts.

TABLE S1 Primers used in this study.
