# Supplementary figures and images for: At5g19540 Encodes a Novel Protein That Affects Pigment Metabolism and Chloroplast Development in Arabidopsis thaliana
Source: Front Plant Sci. 2017 Dec 19;8:2140. doi: 10.3389/fpls.2017.02140 (PMC5742152; doi:10.3389/fpls.2017.02140)

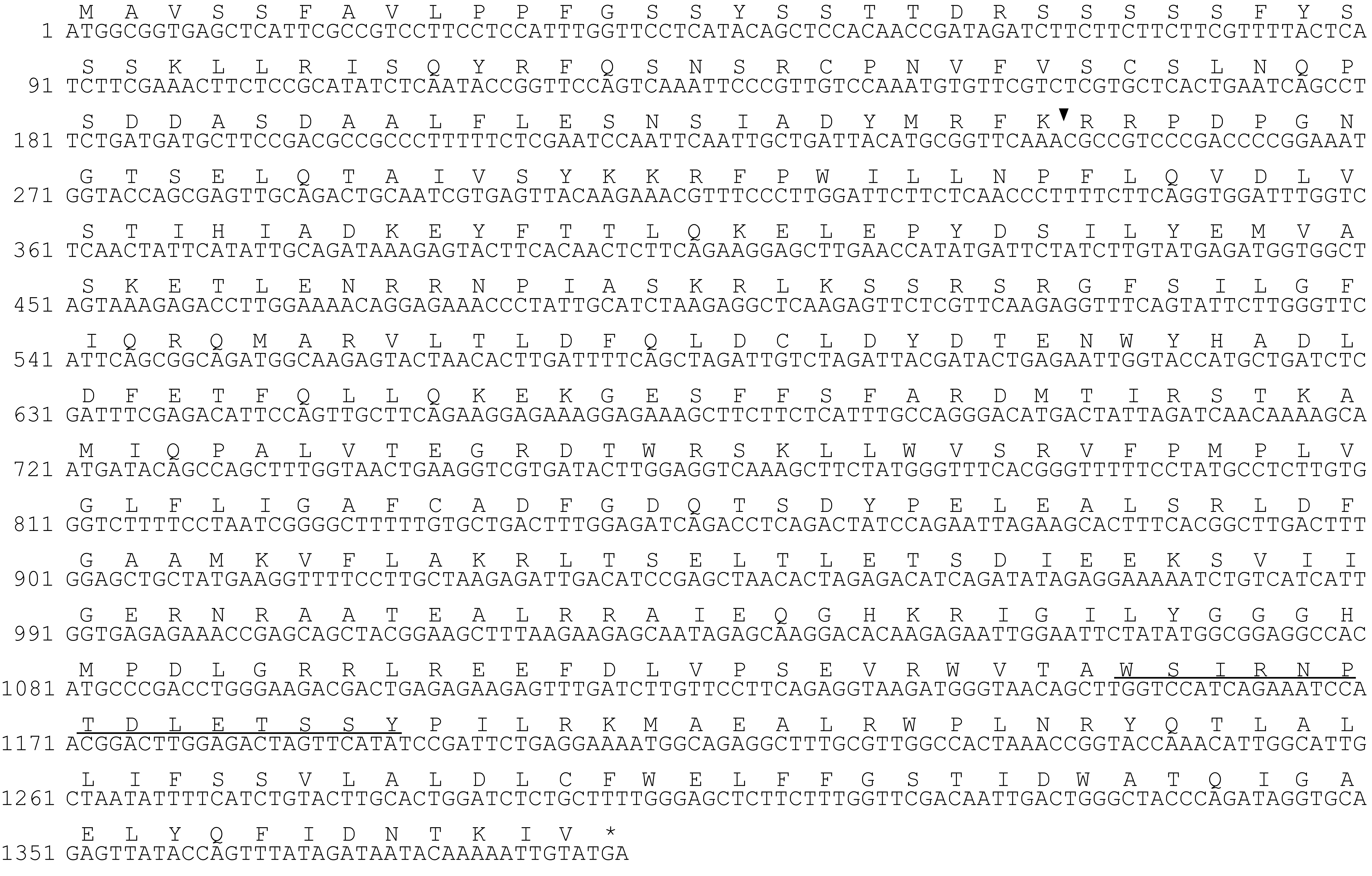

Supplement: FIGURE S1 — Nucleotide and deduced amino acid sequence of DY1. Predicted cleavage site after the chloroplast transit peptide is indicated by a black arrow. Peptide fragment used for raising antiserum against DY1 is underlined. [file Image_1.TIF]

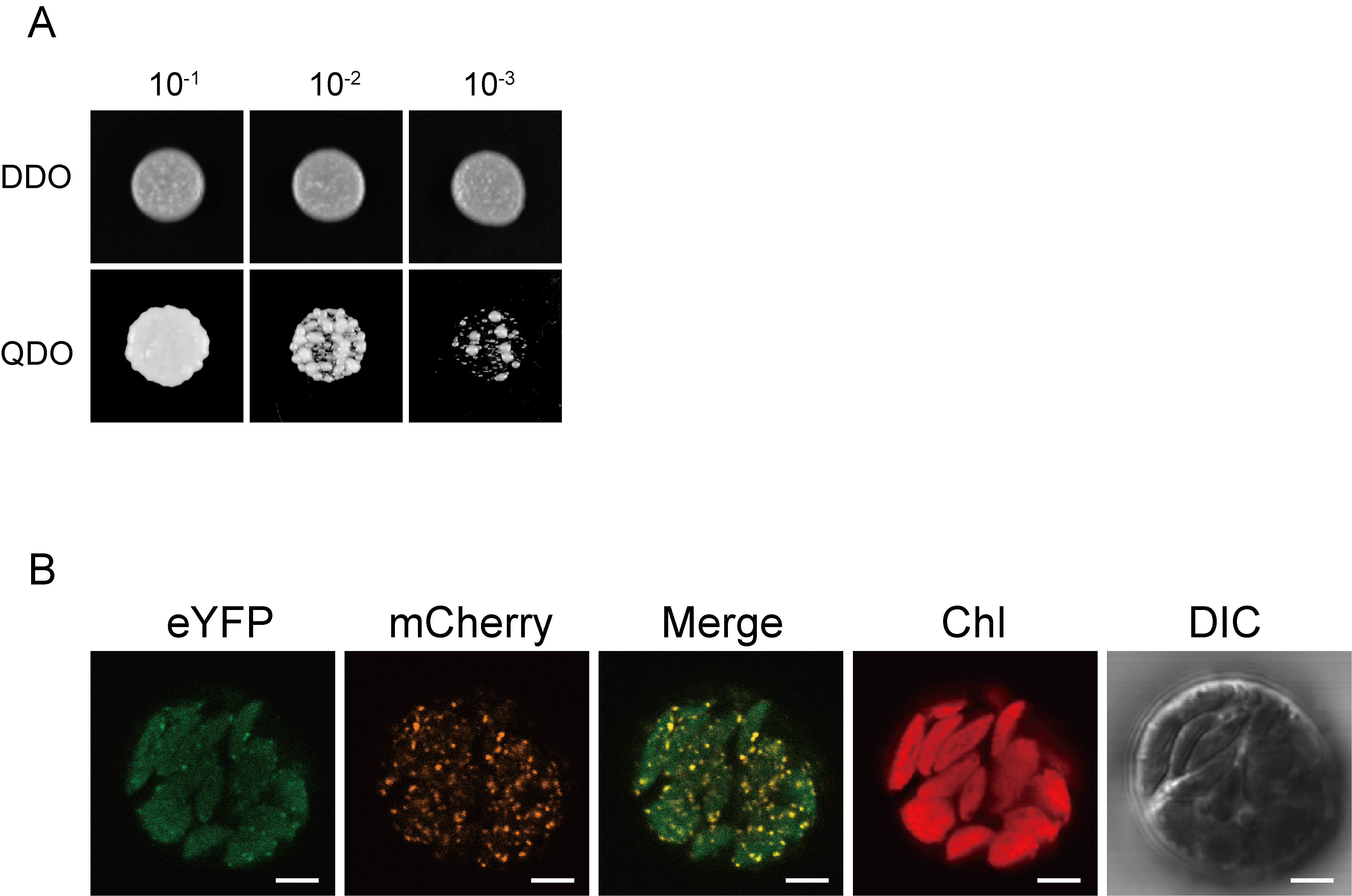

Supplement: FIGURE S2 — DY1 interacts with a RAB GTPase homolog protein At4g20360. (A) Yeast two-hybrid analysis of the interaction between DY1 and At4g20360. DY1 and At4g20360 were cloned into pDEST32 and pDEST22, respectively. Yeast AH109 cells were co-transformed with a combination of these plasmids, and plated onto non-selective (SD/-Leu/-Trp, DDO) and selective (SD/-Leu/-Trp/-His/-Ade, QDO) plates in series dilution. (B) At4g20360 also localizes in chloroplasts. [file Image_2.TIF]
