# Supplementary material for: At5g19540 Encodes a Novel Protein That Affects Pigment Metabolism and Chloroplast Development in Arabidopsis thaliana
Source: Front Plant Sci. 2017 Dec 19;8:2140. doi: 10.3389/fpls.2017.02140 (PMC5742152; doi:10.3389/fpls.2017.02140)
Supplement: TABLE S1 — Primers used in this study. [file Table_1.PDF]

Supplemental Table S1. Primers used in this study.

Primer	Sequence (5'-3')*
LBb1	GCGTGGACCGCTTGCTGCAACT
LBb1.3	ATTTTGCCGATTTCGGAAC
LBa1	ATGGTTCACGTAGTGGGCCATC
DY1-LP	CACAACCGATAGATCTTCTTCTTCTTCGTT
DY1-RP	CGTGAAACCCATAGAAGCTTTGACCTC
DY1-GF	CGCGGATCCTCCACAGGTAATCTAGATTATATATA
DY1-GR	CGCGGATCCGTCGAGAAATTTTATTTACATTTTTT
DY1-HF	CGCGGATCCTCCACAGGTAATCTAGATTATATATA
DY1-ER	CGCGGATCCGTCGAGAAATTTTATTTACATTTTTT
RABE1b-HF	GGGGGATCCATGGCGATTTCGGCTCCA
RABE1b-ER	AGTGGATCCTTCGAGGATCGTCCCAA
DY1-cTP-HF	TTCCTGCAGCCCGGGGGATCCATGGCGGTGAGCTCATTCG
DY1-cTP-ER	CATGCACGAGACGAACACATTTGG
eYFP-HF	ATGTGTTCGTCTCGTGCATGGTGAGCAAGGGCGAGG
eYFP-ER	TCAGTGACTTGTACAGCTCGTCCATGCC
DY1-ΔcTP-HF	CGAGCTGTACAAGTCACTGAATCAGCCTTCTGATGAT
DY1-ΔcTP-ER	GCCCTTGCTCACCATTACAATTTTTGTATTATCTATAAACTGGTATAACTC
mCherry-HF	TAGATAATACAAAAATTGTAATGGTGAGCAAGGGCGAGG
mCherry-ER	CGATCGGGGAAATTCGAGCTCTCACTTGTACAGCTCGTCCATGC
DY1-mature-HF	ATTTACGAACGATAGATGGCGGTGAGCTCATTCG
DY1-mature-ER	TGCCAAAGATGAGTGTCACTTGTACAGCTCGTCCATGC
DY1-Probe-F	CATCTGCCGCTGAATGAACCCAAG
DY1-Probe-R	CTCATTCGCCGTCCTTCCTCCATT
Q-ACT2-F	AAGGCCGAAAATAAAGTTGTAAGAGATAAACCCGC
Q-ACT2-R	GAGCGGAAGAAGATGAGATTGAGGAAGATTCA
Q-DY1-F	TTTCAGTATTCTTGGGTTCATTCAGCGGCA
Q-DY1-R	TCATGTCCCTGGCAAATGAGAAGAAGCTTT
Q-CRD1-F	CCGAGAAAATCGGGTACTGG
Q-CRD1-R	TTCGGTTTGTCTCGATGATGAC
Q-CHLD-F	GGAGCCCTGGTTATCTTTGTG
Q-CHLD-R	GGCTCGACCATCGGTTATC
Q-CHLH-F	CAGGACATGTACTTGAGCCG
Q-CHLH-R	GTACCACTTTGGATTCAGCAGC
Q-CHLI-F	CGGAAGAAGGAGAGCTTAGG
Q-CHLI-R	GCTGCAAGTGCTTTTGCTGC
Q-CHLM-F	GAACCGGTTTGCTCTCGATTC
Q-CHLM-R	GCTCTCCAATTCTCTTTAAGATATCAT
Q-CAO-F	TGGATTTGGCCCGGTGATG
Q-CAO-R	TCTAGTTTCCCGGGTTTTGATATC
Q-FLU-F	GTCTTATGCACCAAGCTTGAGTTAT
Q-FLU-R	GTGATTCCAGAATCTTCACTTTCC
Q-GUN4-F	TCCGAGGTGAAAACAATCTCCC
Q-GUN4-R	CCACTGTTATCATCAGCTGTCG
Q-GRS-F	CTGTGTGCCCTAGACACAC
Q-GRS-R	GAAGCCATGTCAGCTTCAGC

Q-GSA-F	CTGGATTTCGTCTAGCCTATGG
Q-GSA-R	TGCGAAAAAGAATCCAAACATACCG
Q-HemA1-F	GTTGGATCTTGTGTTGGTGAAG
Q--HemA1-R	CTGTTCACGATACCTCGGC
Q-PPOX1-F	CTATCTCGTACCCGAAAGAAGC
Q-PPOX1-R	GGAATGGCTTGAGGCCATAC
Q-PPOX2-F	CTCTCGGTTTTAATCACCACATTC
Q-PPOX2-R	CAACGGGAATGCTTTCCTCC
Q-PORA-F	GAACTCCGATTATCCATCAAAACG
Q-PORA-R	GAGGAATATGCTCTCTGAACAAAC
Q-PORB-F	CCTTCAAAGCGTCTCATCATCG
Q-PORB-R	GGCACGGAAGAGAGGAATGT
Q-PORC-F	CAAAACGTATGATCATCGTAGGATC
Q-PORC-R	AAGCCGAAACAGCGGTATGTG
Q-CLH1-F	GATCCAGTCGCAGGAACTAAC
Q-CLH1-R	CCACCTACAAAGCTCCTCATC
Q-CLH2-F	GGTACTCCTCGAATCTAAAGATC
Q-CLH2-R	CAATAAGAACTCTTCCCTCTAATCC
Q-PAO-F	GAAAGACCCGTTCCATCGTTTG
Q-PAO-R	TGGAAGGGAGAGGTTGGTTAG
Q-RCCR-F	GTGGTCGAACTAATACAGAGC
Q-RCCR-R	CTTCTTCTTCCTTCACACACC
Q-ABA2-F	GGAGGAGCCACAGGGATAGGTGAGAGCAT
Q-ABA2-R	CCATGGATGAAAAAAGCCGTCTCCTTGGAC
Q-ABA4-F	ATTAGATTAGATCATCGTTGGAGCTTCATTGGAGG
Q-ABA4-R	CGGTAGTTCCAACCGCAAATACACTGCTTG
Q-AAO3-F	ATCCATCAACGACCTTACTTGAGTTCTTGCGA
Q-AAO3-R	TTCGTGTTTCCAAGACCTTCAGATGTAGTAATGGA
Q-BCH2-F	TCTTTCTCCGCAAACCACCCTATATCCACC
Q-BCH2-R	TGCTTTCTGGTTTGTTGTCATCGTCCATG
Q-CRTISO-F	GAAGCTGCCTCGTATGGTTACAGTGAAATCCG
Q-CRTISO-R	ACAGCTAGCTGAGTCGCAGCAACTAATCCAC
Q-LYC-F	TCTTTCTGGGAAGAAGATAATTGTCTCCATCTCCA
Q-LYC-R	CCCCCAAAATCGAAGAAAACACCAAAAGGATA
Q-NCED3-F	GAAAAATGGCTTCTTTCACGGCAACGG
Q-NCED3-R	AACATTGAGCTTACGTGTGACACGACTGGC
Q-PDS-F	GGAGTACTGCTGGTCCTTTGCAGGTAGTTTGTG
Q-PDS-R	CCAGCACCAGCAATTACACTTTCAAAGGCTT
Q-PSY-F	GGGTTGCTACTTCTTCTCTAAATCCAGACCCT
Q-PSY-R	TCGGTTCCTTACAAAAGAAGAACTCCAAGTTGGTA
Q-VDE-F	TTCTCGGCTCTGTCTCTGTCTCTCTTCTTCC
Q-VDE-R	GCCAAGCCTACCAATACCATCATCACTTGAGAAA
Q-ZDS-F	TAACAGAGCGATTGCTCTTATCTCAGTTGATTGGC
Q-ZDS-R	GCGTTAACACTCATGTCGGAAACATCGGAG
Q-ZEP-F	GTTTTCCGGAAAACCCGGAGGAGTATCTG
Q-ZEP-R	CCACCTCCGGCAACTAAAACCCTCGATT
Q-LHCA1-F	gtcgTTCAGAATGGCTGCTCACTGGATGC
Q-LHCA1-R	GCCCATCTACAGTGGATGAGCTCTGACTCTTTGTA
Q-LHCA2-F	GTGCTTCTTCTGCCATCGCTGCCAT

Q-LHCA2-R	TGCTTCCCGGGAACCAGATTGGTCTATCT
Q-LHCA3-F	TCTCTTGGTGACAGAAGAAAAGAGTTGAGGAACAG
Q-LHCA3-R	GCTTTAACAACAAAGGAACTCTTCTTCTGTGAGGC
Q-LHCA4-F	ACAAAATCCCCTCCAAGTCTTATTTCTTCACAACC
Q-LHCA4-R	GCCGAGGCATGAGTAGTGACAGTAGCCATTAT
Q-LHCB1-F	AGGCCTTCGCTGAGTTGAAGG
Q-LHCB1-R	GCCTCTACAACGGAGTGAACC
Q-LHCB2-F	CATTGAAGGCTACAGAATCGGAGG
Q-LHCB2-R	CTCACCTGCATCTGTGAAACAAAATC
Q-LHCB3-F	GCATCAACGGTCTTGATGGTGTTG
Q-LHCB3-R	GACTTCTTCTGTGCATAGCAAATAGAAG
Q-LHCB4-F	GTTCCTGAGTGGTACGATGCTG
Q-LHCB4-R	GCCAACCTCCCGTTTGCTAG
Q-LHCB5-F	CACCAACGGATTGGATTTCGAGG
Q-LHCB5-R	GTCAAAAGGAGTTCCAATCGAACAAAG
Q-LHCB6-F	AGCAAACGATGGGTCGATTTCTTC
Q-LHCB6-R	TATCTACAGAATCAAAAACATCATGCCG

*Restriction sites are underlined.
